# A pretreatment 15-gene signature predicts non-pCR in response to neoadjuvant chemotherapy in breast cancer and reveals a pyruvate–*CCND1*–*MET* therapeutic vulnerability

**DOI:** 10.3389/fonc.2026.1829672

**Published:** 2026-08-06

**Authors:** Qiang Li, Jinhao Chen, Wei Yu, Xun Li, Yuhua Deng

**Affiliations:** 1 Department of Pulmonary and Critical Care Medicine (PCCM), Taikang Tongji (Wuhan) Hospital, Wuhan, Hubei, China; 2 Taikang Clinical College of Wuhan University, Wuhan University, Wuhan, Hubei, China; 3 Hubei Key Laboratory of Tumor Microenvironment and Immunotherapy, College of Basic Medical Science, China Three Gorges University, Yichang, China; 4 Center for Gene Diagnosis and Department of Clinical Laboratory Medicine, Zhongnan Hospital of Wuhan University, Wuhan, Hubei, China; 5 Southern University of Science and Technology Affiliated Foshan Hospital (The First People’s Hospital of Foshan), Foshan, Guangdong, China

**Keywords:** breast cancer, MET signaling pathway, NAC, non–pathologic complete response, pyruvate metabolism

## Abstract

**Background:**

In breast cancer (BC), neoadjuvant chemotherapy (NAC) improves surgical options, and pathologic complete response (pCR) predicts better outcomes. Patients with residual disease (non-pCR) have higher recurrence risk, yet robust pretreatment biomarkers remain limited.

**Objective:**

To develop a pretreatment transcriptomic predictor of non-pCR and connect the predictive signal to an interpretable metabolic–transcriptional–signaling mechanism with potential therapeutic actionability.

**Methods:**

Three Gene Expression Omnibus (GEO) pretreatment bulk-expression cohorts were analyzed (GSE25066, GSE20194, GSE32646). Differentially expressed genes (DEGs) were integrated and a least absolute shrinkage and selection operator (LASSO) logistic model (trained on GSE25066) generated a 15-gene risk score (RS), externally validated in GSE20194 and GSE32646. A hospital clinical cohort provided paired samples and serum metabolomics focusing on glycolysis-related metabolites. Functional assays and RNA-seq tested pyruvate effects and MET inhibition (capmatinib). Single-cell RNA-seq plus inferCNV was used to localize key signals and infer a regulatory axis.

**Results:**

The 15-gene RS showed consistent discrimination for non-pCR (AUC: 0.823 training; 0.809 and 0.876 validations). Non-pCR tumors exhibited enriched cell-cycle programs (E2F/G2M), inflammatory signaling (TNFα/NF-κB), and estrogen-response pathways. Multi-omics highlighted *CCND1* as a central hub; serum pyruvate was elevated in non-pCR and positively correlated with CCND1. Pyruvate enhanced malignant phenotypes and induced transcriptomic changes centered on *MET* signaling; capmatinib attenuated these effects. Single-cell analyses showed highest CCND1 expression in epithelial/malignant compartments and supported a *YAP1–HIF1A/ARNT–MET* transcriptional axis linking pyruvate-associated cues to MET activation.

**Conclusions:**

A pretreatment 15-gene RS enables risk stratification for NAC non-pCR, and mechanistic evidence supports a pyruvate–*CCND1–MET* vulnerability potentially addressable by MET-targeted combination strategies.

## 1 Introduction

Neoadjuvant chemotherapy (NAC) is widely used in breast cancer (BC) to reduce tumor burden, increase the feasibility of breast-conserving surgery, and inform subsequent treatment decision (1). In clinical practice, pathologic complete response (pCR) is often regarded as a desirable endpoint, as patients who achieve pCR tend to have better long-term outcomes (2). By contrast, patients with residual disease after NAC (non-pCR) face a higher risk of recurrence and poorer prognosis, and they often require more intensive postoperative therapy and follow-up (3). A major limitation, however, is that response is usually confirmed only after NAC through pathologic assessment (4). Before treatment, there remains a lack of reliable biomarkers that can consistently and accurately stratify patients (5). As a result, some patients with limited expected benefit still receive standard regimens, experience toxicity, and may miss an opportunity for a more appropriate alternative treatment (6). Existing indicators—such as imaging-based tumor shrinkage, molecular subtype, changes in Ki-67, or immune infiltration—vary across populations and testing platforms, which limits their generalizability, practicality, and interpretability (7). Therefore, there is an urgent need for predictive tools that can be applied to pretreatment samples. All enrolled BC patients received standard neoadjuvant chemotherapy in clinical practice.

Emerging evidence suggests that non-pCR is not a uniform phenotype caused by a single resistance route (8). Rather, it appears to reflect a complex state of reduced treatment sensitivity shaped by multiple biological programs (9). Compared with pCR tumors, non-pCR tumors often show coordinated increases in gene expression patterns linked to cell division and proliferation (for example, *E2F*-associated programs and the G2/M checkpoint), alongside shifts in inflammatory signaling (such as TNFα/NF-κB) and hormone-response pathways (including estrogen response) (10). Together, these changes suggest that tumors may endure chemotherapy by sustaining proliferative capacity while reshaping stress- and immune-related signaling to support survival and regrowth (11). This composite state also implies that single-gene or single-pathway markers may be insufficient to capture the system-level nature of resistance, and that bulk transcriptional signals can be confounded by intratumoral heterogeneity and differences in microenvironmental composition (12). Consistent with this view, integrative multi-omics analyses highlight *CCND1* as a central hub associated with the non-pCR phenotype, suggesting roles beyond cell-cycle control and pointing to potential coupling with metabolic shifts and pro-survival signaling networks that stabilize resistance (13). Taken together, linking prediction to mechanism can help address three practical questions: who is likely to respond poorly, why reduced sensitivity arises, and whether actionable vulnerabilities exist.

We collected tumor samples before chemotherapy and retrospectively grouped patients according to their post-NAC pathologic outcomes. By integrating multiple transcriptomic cohorts of NAC-treated patients, we used LASSO regression to derive a parsimonious 15-gene risk score (RS). The score showed consistent discriminatory performance in independent datasets, supporting its potential utility for pretreatment prediction of NAC response. Importantly, we then connected this predictive signal to a biological rationale using multi-omics and functional evidence. Serum metabolomics indicated glycolysis-related metabolic shifts in non-pCR patients, including elevated pyruvate levels, and pyruvate abundance was strongly correlated with *CCND1* expression. *In vitro*, pyruvate increased cell migration and invasion and induced broad gene-expression changes, accompanied by activation of *MET*-related signaling. Pharmacologic inhibition of MET attenuated the pyruvate-enhanced malignant phenotypes, suggesting a potentially druggable vulnerability. Single-cell analyses further showed enrichment of CCND1 in epithelial and tumor cell populations and pointed to a putative regulatory axis linking metabolic cues to receptor signaling (*YAP1–HIF1A/ARNT–MET*). This axis offers a testable route by which metabolic alterations could be converted into pro-tumor signaling outputs. Overall, we propose an integrated framework that couples pretreatment prediction with mechanistic closure, connecting metabolic change, transcriptional regulation, and pro-survival signaling in NAC-resistant BC. This framework provides a basis for clinical risk stratification, rational combination-therapy design, and prospective validation.

## 2 Material and methods

### 2.1 Data acquisition and preprocessing

We obtained BC bulk transcriptomic datasets (GSE25066, GSE20194, and GSE32646) and a single-cell transcriptomic dataset (GSE205472) from the GEO repository. The bulk cohorts included GSE25066 (n = 488; pCR = 99, non-pCR = 389), GSE20194 (n = 278; pCR = 56, non-pCR = 222) and GSE32646 (n = 115; pCR = 27, non-pCR = 88). Inclusion criteria for bulk datasets were: (1) tumor samples collected before NAC; and (2) availability of pathological response data (pCR or non-pCR) for all patients. The single-cell dataset comprised 13 BC samples, including 8 pre-NAC samples (3 pCR and 5 non-pCR) and 5 post-NAC samples (2 pCR and 3 non-pCR). In addition to these public datasets, we collected an independent clinical cohort, which was approved by the Ethics Committee of the First People’s Hospital of Foshan (Ethics Approval Number: FSYYY-EC-ZN-3.0-A02-2024), comprising 20 healthy control blood samples, 12 pCR blood samples, 18 non-pCR blood samples, and paired samples (17 non-pCR tumor samples and 10 pCR tumor samples), the details of which are summarized in **Supplementary Table 1**. All participants provided written informed consent. TPM values, gene expression counts and clinical data for the BC cohort of The Cancer Genome Atlas (TCGA) were downloaded from the TCGA database. Glycolysis-related genes were retrieved from the Gene set enrichment analysis (GSEA) database using the HALLMARK_GLYCOLYSIS gene set. Transcription factor-related datasets with a Relevance score > 16 were acquired from the GeneCards database (https://www.genecards.org/) in **Supplementary Table 2**. All datasets were derived from publicly available or investigator-shared Homo sapiens samples. All patients from the three GEO cohorts and our clinical cohort had invasive BC and received standard NAC. We focused on the common molecular profiles of NAC-resistant BC across different molecular subtypes and did not perform subgroup analyses stratified by ER, PR and HER2 status in this study. All analysis codes are deposited at https://github.com/chenjinhao-nb/CCND1-paper-code for full result replication.

### 2.2 Identifying differentially expressed genes

Based on the RNA-seq count data, differentially expressed mRNAs were identified using the limma R package (version 3.64.3) (14). Differential expression analysis was performed with linear models and moderated t statistics implemented in limma to estimate *P* values for each gene and quantify between-group differences. To control for multiple testing, *P* values were adjusted using the Benjamini–Hochberg false discovery rate procedure. Genes satisfying adjusted *P*-value < 0.05 and |log2(fold change)| > 0.585 were defined as differentially expressed genes (DEGs). Consensus DEGs overlapping across all three transcriptomic cohorts (GSE25066, GSE20194, GSE32646) were retained for subsequent prognostic model construction.

### 2.3 Functional enrichment analysis

We performed Gene Ontology (GO) enrichment analysis using the clusterProfiler R package (version 4.16.0) to identify overrepresented biological processes, cellular components and molecular functions associated with the selected genes (15). Kyoto Encyclopedia of Genes and Genomes (KEGG) pathway enrichment was also conducted with the same package to uncover key signaling pathways. Enrichment terms with an adjusted *P* value < 0.05 were considered significant, and the top GO terms and KEGG pathways were visualized as dot plots generated with ggplot2. To further explore pathway and functional differences between subgroups, we performed gene set enrichment analysis (GSEA) using Hallmark gene sets, the KEGG_MEDICUS subset of the curated collection and GO gene sets obtained from the MSigDB resource (https://www.gsea-msigdb.org/gsea/login.jsp).

### 2.4 Predictive model construction

The non-pCR risk model was constructed using the GSE25066 cohort as the training set and validated in two independent cohorts, GSE20194 and GSE32646. Candidate predictors for model building were the 35 consensus DEGs identified in Section 2.2. Univariate logistic regression was conducted with NAC pathological response (pCR vs non-pCR) as the binary endpoint, and DEGs with *P* < 0.05 were retained as candidate variables for LASSO modeling. These genes were subsequently included in LASSO regression analysis using the glmnet R package (version 4.1.10) (16). The LASSO regression was tuned via 10-fold cross-validation to select the optimal penalty parameter λ = 0.01456118; coefficient trajectories under varying λ values were visualized to yield a 15-gene signature. Based on the median non-pCR RS derived from the model, BC patients were stratified into high-risk and low-risk groups. The predictive performance of the risk model was evaluated using receiver operating characteristic (ROC) curves.

### 2.5 RNA sequencing and transcriptomic data processing

Total RNA was extracted from harvested and washed target cells using the MasterPure Kit. Following poly (A) + mRNA enrichment with oligo (dT) beads, sequencing libraries were constructed using the Vazyme Nano DNA Library Prep Kit. The libraries were sequenced on an Illumina HiSeq2500 platform, generating approximately 81 million paired-end reads per sample. Raw sequencing reads were trimmed for adapters and low-quality bases using Cutadapt. The clean reads were then aligned to the GRCh38 reference genome (GENCODE annotation) using STAR. Duplicate reads were removed with Samtools, and gene-level counts were obtained using featureCounts with strand-specific settings. Gene expression was normalized as Transcripts Per Kilobase Million (TPM). Genes with TPM < 1 across all samples were filtered out prior to downstream analysis to ensure reliability.

### 2.6 Single-cell RNA-seq data preprocessing and inferCNV analysis

All samples were analyzed using the Seurat framework (version 5.3.1), a toolkit for quality control, analysis, and exploration of single-cell RNA sequencing data. Seurat aims to enable users to identify and interpret sources of heterogeneity from single-cell transcriptomic measurements, and to integrate diverse modalities of single-cell omics data (17). Stringent quality-control filters were applied to retain genes detected in at least three cells and cells expressing at least 200 genes, with thresholds set as 200 < nFeature_RNA < 6,000 and mitochondrial gene proportion (percent.mt) < 10%. Batch effects were corrected using the Harmony integration algorithm. We then used Seurat’s RunPCA function to perform principal component analysis (PCA) on the top 3,000 highly variable genes, yielding 50 principal components (PCs). For downstream analyses, UMAP embedding was calculated based on the first 30 PCs to generate two-dimensional cell visualizations; cell clustering was conducted with Seurat’s FindClusters function on the Harmony-corrected first 30 PCs at a resolution of 0.5. Single-cell copy number variation (CNV) analysis for target cell populations was performed using the inferCNV R package (version 1.24.0) with a cutoff of 0.1 (18). The raw single-cell gene count matrices, cell type annotations, and genomic coordinates of genes were used as inputs, and T cells were set as the normal reference population.

### 2.7 Untargeted metabolomic analysis

For serum metabolites, the samples were analyzed using Liquid Chromatograph Mass Spectrometer (LC-MS) at the platform of Orbitrap Exploris 480 (Thermo Scientific, MA, USA). All processes were carried out according to standard protocols. Initially, quality control samples were used to validate equipment stability and the reliability of experimental procedures. Blank samples were also employed to minimize noise interference. The supernatant, diluted with 80% methanol in sterile water (LC-MS Grade, Merck, Germany, CAS: 7732-18-5), was centrifuged again under identical conditions. The final supernatant was then subjected to LC-MS analysis (Q ExactiveTM HF-X, Thermo Fisher, USA) with the following parameters: m/z: 100 ~ 1500, Spray Voltage: 3.2 kV, Sheath gas flow rate: 40 arb, Aux gas flow rate: 10 arb, Capillary temp: 320 °C, Funnel RF level: 40, Aux gas heater temp: 350 °C, Polarity: positive and negative (19).

### 2.8 Cell cultures

The MDA-MB-231 human BC cell line (#CL-0103) was purchased from iCell (Shanghai, China). Cells were cultured in Dulbecco’s Modified Eagle’s Medium (DMEM) supplemented with 10% fetal bovine serum (FBS) and 1% penicillin-streptomycin. The cells were maintained at 37 °C in a humidified atmosphere containing 5% CO_2_.

### 2.9 Cell transfection

To investigate the function of *CCND1*, we knocked down its expression using RNA interference (RNAi). In this study, three distinct small interfering RNAs (siRNAs) targeting *CCND1* and a non-targeting scrambled siRNA were used, and their sequences are shown in Extended Data **Supplementary Table 3**. The storage concentration of all siRNAs was 20 μmol/L. To ensure efficient gene knockdown, the three *CCND1*-targeting siRNAs were mixed at a ratio of 1:1:1. Cells were transfected with mixed si-*CCND1* or scrambled control siRNA at a final concentration of 100 nM for 48 h. Cells were then harvested for Western blot analysis to verify *CCND1* knockdown efficiency.

### 2.10 Cell scratch assay

MDA-MB-231 cells were seeded into 6-well plates at a density of 5 × 10^5^ cells/well. When cells reached approximately 70% confluence, cells were transfected with si-*CCND1* or scrambled control siRNA. For groups treated with pyruvate or the *MET* inhibitor capmatinib, the corresponding compounds were supplemented immediately after transfection. Following 48 h incubation to achieve stable CCND1 knockdown and sustained drug treatment, a straight scratch was created in each well using a sterile 200 μL pipette tip. The culture medium was subsequently replaced with serum-free medium. Cell migration images were captured at designated time points under an Olympus IX71 inverted microscope at 100× magnification: 0, 12, and 24 h for siRNA-only groups; 0, 12, 24, and 48 h for compound-treated groups. The width of the scratch gap was quantified using ImageJ software. All experiments were performed in three independent biological replicates (https://imagej.nih.gov/ij).

### 2.11 Transwell assay

Cell invasion capability was assessed using a Transwell assay. A suspension of 5 × 10^4^ cells in FBS-free medium was plated into the upper chamber of Transwell 24-well plates, which featured 8 μm pore filters (Corning). The lower chamber was filled with 10% FBS medium supplemented with pyruvate, the *MET* inhibitor capmatinib, or vehicle controls. Following a 48 h incubation period, the upper chamber and medium were carefully removed. The cells that had migrated to the lower surface of the membrane were fixed and stained with 0.5% crystal violet for 30 minutes at room temperature. The extent of migration was quantified by counting the number of cells per field of view using ImageJ software (https://imagej.nih.gov/ij).

### 2.12 Western blot

Total protein was extracted from cultured cells, and the protein concentration was determined using a BCA protein assay kit. Cell lysates were mixed with 5×SDS loading buffer at a volume ratio of 1:4, and the mixture was denatured by heating at 98 °C for 10 min. Equal amounts of protein (30 μg per sample) were separated by 10% SDS-polyacrylamide gel electrophoresis (SDS-PAGE) and then electrotransferred onto polyvinylidene fluoride (PVDF) membranes (Servicebio, China, Cat#G6015-0.45). After blocking the membranes with 5% non-fat milk for 1 h at room temperature, the blots were incubated with primary antibodies against *CCND1, MET, YAP1, HIF1A* and *GAPDH* at 4 °C overnight, followed by incubation with HRP-conjugated anti-rabbit or anti-goat secondary antibodies for 1 h at room temperature. Immunoreactive bands were visualized using an enhanced chemiluminescence (ECL) western blotting substrate, and the signals were captured with a Syngene bioimaging system (Gene Gnome, Hong Kong, China). Image acquisition and densitometric analysis of the blots were performed using ImageJ software. The integrated density value (IDV) of each target protein band was normalized to that of the internal reference *GAPDH*, and the IDV ratio (target protein/GAPDH) was calculated for quantitative analysis of protein expression levels.

### 2.13 Luciferase reporter assay

The wild-type promoter sequences of *MET, HIF1A* and *ARNT* were synthesized, and their corresponding mutant promoter sequences (with binding site mutations) were also constructed. All sequences were subcloned into luciferase reporter vectors separately and verified by Sanger sequencing. The constructed luciferase reporter vectors were co-transfected into 293T cells with either *YAP1* overexpression plasmids or empty vector controls. The relative luciferase activity of the transfected cells was determined using a Dual-Luciferase Reporter Assay Kit in strict accordance with the manufacturer’s instructions, so as to verify the transcriptional regulatory effect of *YAP1 on MET, HIF1A* and *ARNT*.

### 2.14 Statistical analysis

The data in this study were presented as mean ± standard error of the mean (SEM), and statistical analyses were performed using GraphPad Prism (v9.5). Depending on the types of data, statistical analysis between two groups was conducted using either the Wilcoxon rank-sum test or Student’s *t* test. For multi-group comparisons, one-way analysis of variance (ANOVA) or Dunnett’s test was employed. Correlation analyses were carried out using Pearson’s rho statistics. In all boxplots, the center lines represent the median, and the box edges represent the first and third quartiles. In the figures, p < 0.05 indicates statistical significance (*p < 0.05, **p < 0.01, ***p < 0.001, ****p < 0.0001), while “ns” indicates no significant difference. The p-value for PCA was calculated from a *Wilcoxon* rank-sum test on the samples’ projection onto PC1.

## 3 Result

### 3.1 Development and validation of a 15-gene signature for predicting NAC outcomes in BC

**Figure 1:**
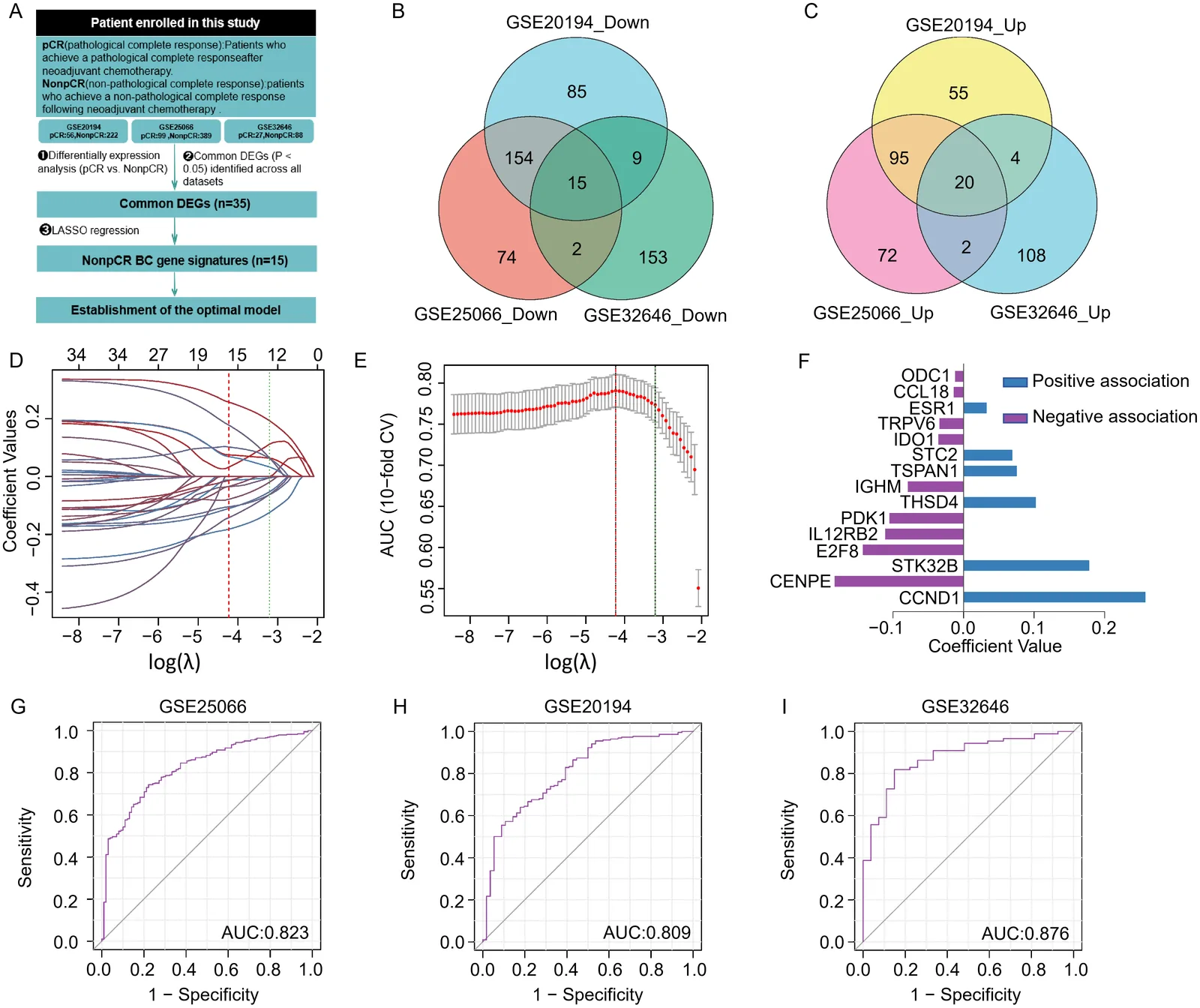
Identification of non-pCR-related DEGs and construction of a RS model. **(A)** Patients were enrolled based on their response to NAC: those with pCR versus those with non-pCR. **(B, C)** Venn diagram depicting the 35 common DEGs among GSE20194, GSE25066, and GSE32646 datasets, including 15 downregulated and 20 upregulated genes. **(D, E)** LASSO regression analysis and the selection of the optimal lambda (λ) value. The red dashed line indicates the optimal λ value (0.01456118). **(F)** Genes selected by the LASSO model and their association with non-pCR. Genes showing a positive association with non-pCR (*CCND1*, *STK32B*, *THSD4*, *TSPAN1*, *STC2*, and *ESR1*) and genes showing a negative association (*CENPE*, *E2F8*, *IL12RB2*, *PDK1*, *IGHM*, *TRPV6*, *CCL18*, *ODC1*) are displayed. **(G–H)** Predictive potency was determined via ROC analysis in the training set (GSE25066; **G**) and validation sets (GSE20194; **H**, GSE32646; **I**).

We stratified patients by their response to NAC into a pathological complete response (pCR) group and a residual disease (non-pCR) group. To identify transcriptomic determinants of NAC response, we analyzed three independent gene-expression datasets (GSE25066, GSE20194, and GSE32646) from NAC-treated BC patients; clinical characteristics are summarized in **Supplementary Table 1**. Venn analysis of DEGs across the three datasets identified 35 consensus genes that discriminate pCR from non-pCR samples (**Figures 1A–C**). Among these genes, 20 were upregulated and 15 were downregulated in non-pCR compared with pCR.

**Figure 2:**
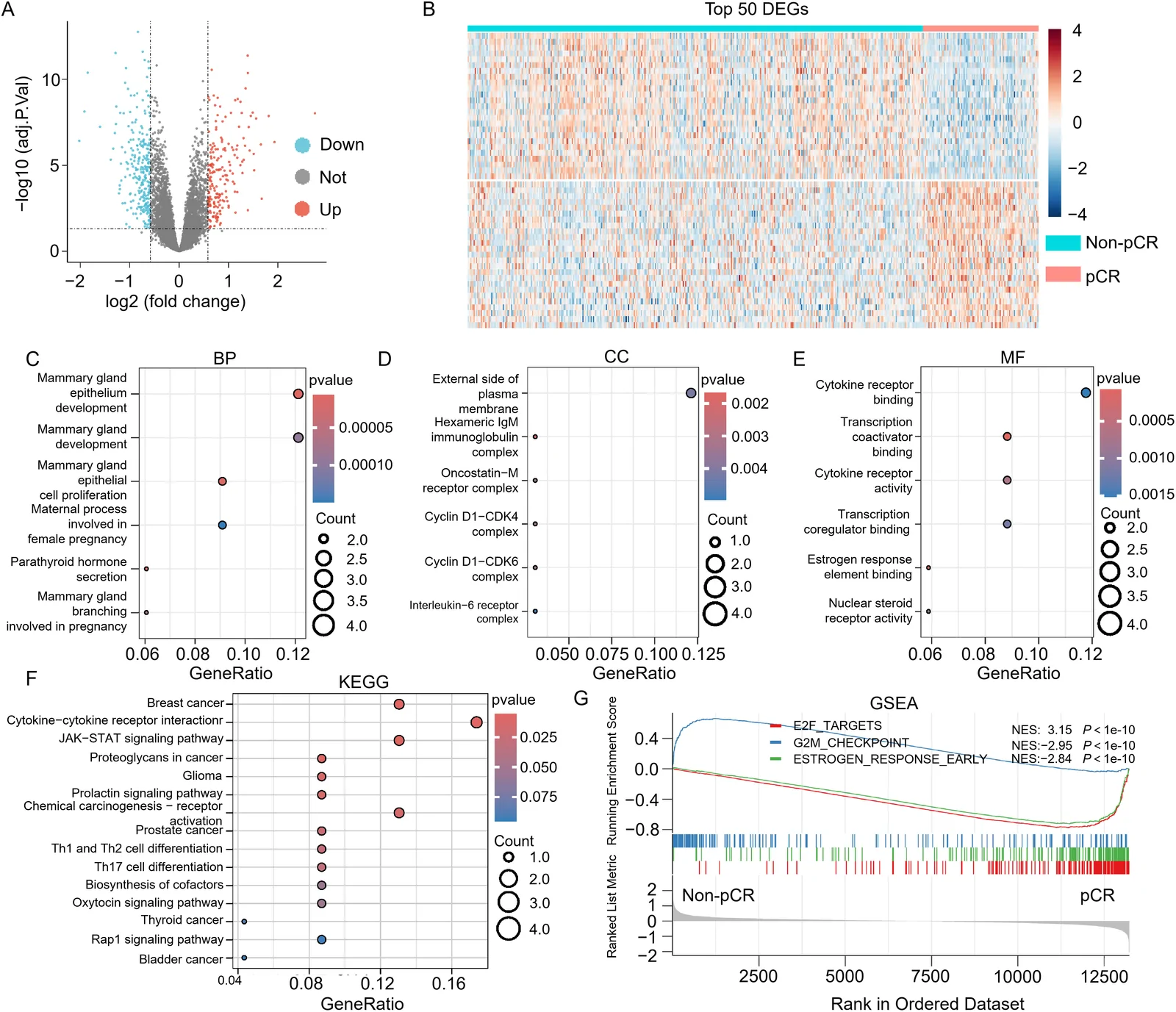
Transcriptomic and functional characteristics of glycolysis-defined subtypes. **(A)** Volcano plot displaying DEGs between non-pCR and pCR samples. **(B)** Heatmap of the expression patterns of the identified DEGs across non‑pCR and pCR samples. **(C–E)** GO enrichment analysis of the DEGs, showing significant terms in the biological process **(C)**, cellular component **(D)**, and molecular function **(E)** categories. **(F)** Significantly enriched KEGG pathways among the DEGs. **(G)** GSEA reveals hallmark pathways enriched in non-pCR and pCR samples.

Prior to signature construction, candidate predictors for model building were the 35 consensus DEGs derived from three datasets GSE25066, GSE20194 and GSE32646. Univariate logistic regression was conducted with NAC pathological response (pCR vs non-pCR) as the binary endpoint, and DEGs with P < 0.05 were retained as candidate variables for subsequent LASSO regression. To derive a predictive signature from these candidate genes, we trained a LASSO model using GSE25066 as the training cohort. This procedure yielded a parsimonious 15-gene signature; the corresponding regression coefficients are provided in **Supplementary Table 4**.

Using the LASSO model trained on GSE25066, we estimated the probability of non-pCR for each sample in all cohorts. ROC analysis showed good discrimination, with AUCs of 0.823 for the training cohort (GSE25066), 0.809 for the validation cohort GSE20194, and 0.876 for the validation cohort GSE32646 (**Figures 1D–F**), supporting the model’s generalizability. Univariate logistic regression further indicated that the RS was consistently associated with response status across cohorts (**Figures 1G–I**).

### 3.2 Transcriptomic profiling delineates a proliferative and inflammatory landscape in treatment-resistant BC

**Figure 3:**
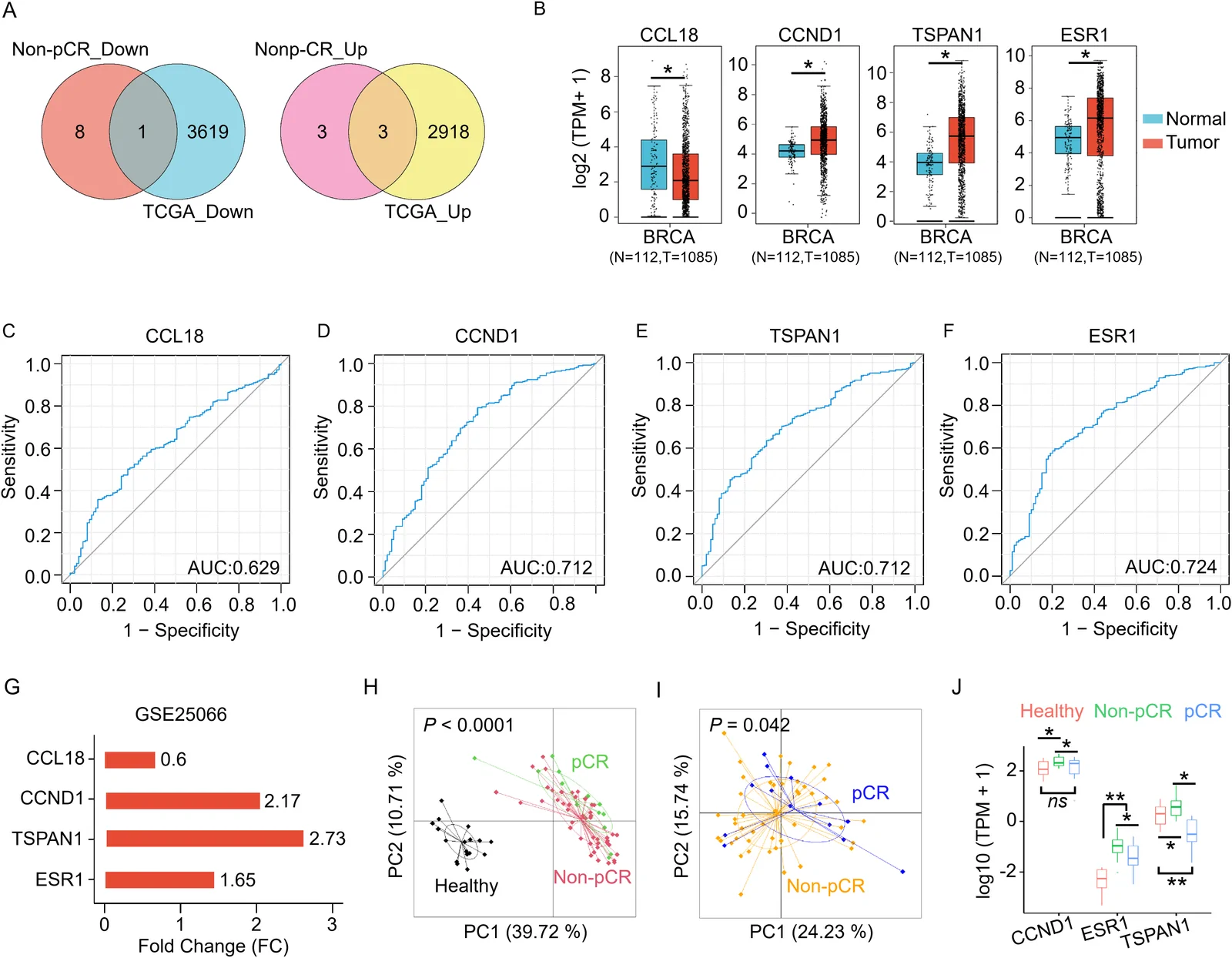
CCND1 was the most significantly upregulated gene in the non-pCR group compared with the pCR group. **(A)** Venn diagram depicting the 4 common DEGs, including 1 downregulated and 4 upregulated genes. **(B)** The mRNA expression levels of *CCL18*, *CCND1*, *TSPAN1* and *ESR1* in the TCGA dataset. **(C–F)** ROC curve analysis of the four-gene risk model for the GSE25066 dataset. **(G)** Fold change of the four genes in the GSE25066 dataset. **(H–I)** PCA analysis in our institutional cohort. **(J)** *CCND1*, *ESR1* and *TSPAN1* Expression in our institutional cohort. Statistical significance was determined by multivariate logistic regression analysis for forest plots and two-tailed Student’s t-test for box plots: * *P* < 0.05, ** *P* < 0.01.

All differential expression analyses were performed on the training dataset GSE25066. This cohort was chosen due to its large sample size and well-balanced pCR/non-pCR subgroups, which enhanced the robustness of the identification of DEGs. Following the standard pipeline for signature development, GSE25066 was used for the screening of DEGs, feature selection and LASSO model construction, while the other two independent cohorts were exclusively applied for external validation to assess the generalizability of the 15-gene signature and prevent overfitting. To characterize molecular differences between patients with residual disease and those with a pathological complete response, we performed differential expression analysis comparing non-pCR with pCR samples. We identified 443 significant DEGs, including 198 upregulated and 245 downregulated genes in non-pCR relative to pCR (**Figures 3A, B**).

GO enrichment analysis highlighted biological themes associated with non-response. Enriched biological processes included regulation of epithelial cell proliferation and mammary gland epithelium development (**Figure 3C**). For the cellular component category, upregulated DEGs were enriched in complexes involved in cell-cycle progression (e.g., the cyclin D1–CDK4 complex) and in components related to immune receptor activity (**Figure 3D**). In the molecular function category, enriched terms were mainly related to transcriptional regulation (e.g., RNA polymerase II–specific transcription coactivator activity) and steroid hormone signaling (e.g., nuclear steroid receptor binding) (**Figure 3E**).

KEGG pathway enrichment analysis indicated that DEGs in the non-pCR group were significantly enriched in oncogenic and immunomodulatory pathways, including BC, JAK–STAT signaling, and Cytokine–cytokine receptor interaction (**Figure 3F**). These results are consistent with increased proliferative signaling and an immunosuppressive tumor microenvironment in non-pCR, which may contribute to poor response to NAC.

Consistently, GSEA showed positive enrichment of hallmark gene sets in non-pCR, including cell-cycle programs (*E2F*_TARGETS, G2M_CHECKPOINT), inflammatory signaling (TNFA_SIGNALING_VIA_NFKB), and estrogen response (**Figure 3G**).

Although several individual enriched pathways have been reported in previous BC NAC studies, our overall results possess distinct novelty. The present enrichment and GSEA analyses were based on the unique DEGs screened from our training dataset, instead of reusing published datasets. Different from prior work that only described separate pathway alterations in chemotherapy-resistant tumors, we further linked these pathway signatures to our predictive 15-gene signature, the central hub gene CCND1, and downstream metabolic signaling networks. This systematic correlation analysis and mechanistic exploration represent the original contributions of our study.

In summary, integrated transcriptomic profiling reveals a coordinated molecular program in non-pCR patients, marked by upregulation of cell-cycle regulators, pro-tumorigenic inflammatory signaling, and developmental pathways. Collectively, these alterations extend beyond metabolic reprogramming and suggest a multifactorial biological basis for poor response to chemotherapy.

### 3.3 Multi-omics integration identifies CCND1 as a central hub gene in chemotherapy resistance

To identify candidate molecular determinants of differential response to NAC in BC, we intersected transcriptomic differences between non-pCR and pCR cohorts with a set of tumorigenesis-related genes curated from TCGA. This intersection yielded four core response-associated genes: *CCL18* was downregulated, whereas *CCND1, TSPAN1*, and *ESR1* were upregulated in non-pCR tumors (**Figure 2A**).

Independent validation using GEPIA showed that all four genes were significantly differentially expressed in breast tumors compared with normal breast tissue (**Figure 2B**). In single-gene ROC analyses, each gene demonstrated discriminative ability, with AUCs of 0.629 (*CCL18*), 0.712 (*CCND1*), 0.712 (*TSPAN1*), and 0.724 (*ESR1*) (**Figures 2C–F**).

In the GSE25066 dataset, *CCND1* (fold change = 2.17) and *TSPAN1* (fold change = 2.73) were markedly overexpressed in non-pCR samples, consistent with their association with poor response to NAC (**Figure 2G**). PCA of our institutional cohort (17 normal breast tissues, 46 non-pCR tumors, and 13 pCR tumors) showed separation among the three groups, supporting the biological relevance of these genes (**Figures 2H, I**). Notably, in our cohort, *CCND1* was the most significantly upregulated gene in non-pCR relative to pCR, suggesting it may contribute to differential treatment outcomes (**Figure 2J**).

Collectively, these multilayer analyses define a concise four-gene signature that discriminates non-pCR from pCR BC and may have clinical utility. Among these genes, *CCND1* is a promising candidate for predictive biomarker development and a potential therapeutic target.

### 3.4 A glycolysis-driven pyruvate-CCND1 axis underlies treatment resistance in BC

**Figure 4:**
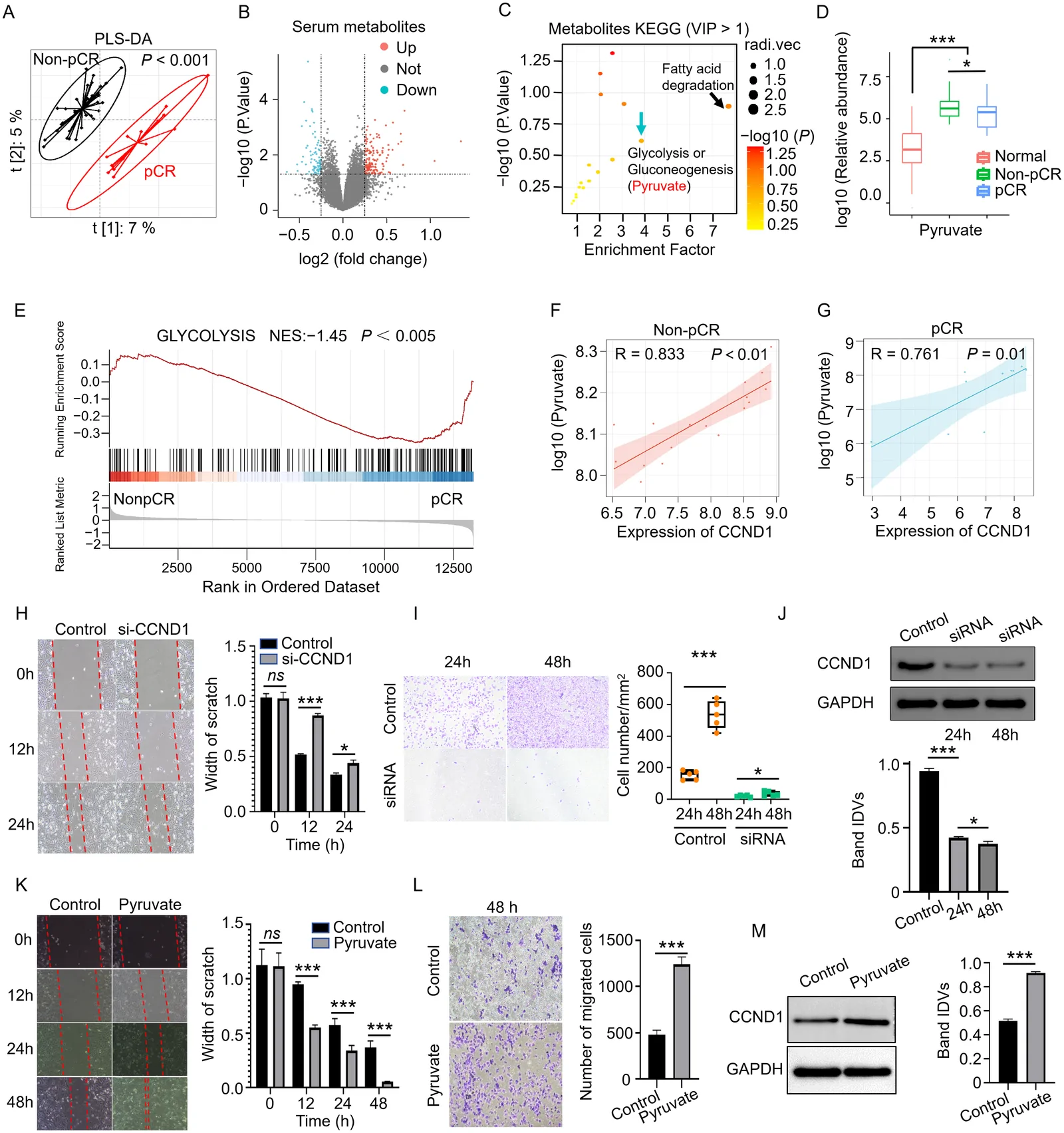
Glycolysis-Driven Pyruvate–CCND1 axis promotes tumor migration and invasion. **(A)** PLS-DA analysis of serum metabolomics data. **(B)** Volcano plot showing differentially abundant serum metabolites. **(C)** KEGG pathway enrichment analysis of differential serum metabolites. **(D)** Relative pyruvate abundance across samples from our institutional clinical cohort. **(E)** GSEA of glycolysis signature in paired tumor tissues. **(F, G)** Correlation analysis between pyruvate level and *CCND1* expression. **(H, I)** Representative images and quantitative statistics of wound-healing migration and Transwell invasion assays, demonstrating that CCND1 knockdown markedly suppresses cell migratory and invasive capacities. **(J)** Western blot analysis validating the knockdown efficiency of *CCND1*-targeted siRNA. **(K, L)** Wound-healing and Transwell assays revealing that exogenous pyruvate treatment significantly enhances cell migration and invasion. **(M)** Western blot showing elevated *CCND1* protein expression upon pyruvate stimulation. Results were summarized as mean ± SD of three independent experiments. (* *P* < 0.05; ** *P* < 0.01; *** *P* < 0.001, independent Student’s t-test).

To characterize metabolic differences associated with NAC response, we performed serum metabolomics profiling in 30 BC patients, stratified into non-pCR (n = 18) and pCR (n = 12) groups. We identified 224 differentially abundant metabolites, including 143 increased and 81 decreased in non-pCR compared with pCR (**Figures 4A, B**).

Pathway enrichment analysis of differentially abundant metabolites showed significant enrichment of fatty acid degradation and glycolysis/gluconeogenesis pathways (**Figure 4C**). Pyruvate emerged as a central hub metabolite. Consistent with these findings, serum pyruvate levels were higher in BC patients than in healthy controls and were highest in the non-pCR group (**Figure 4D**). GSEA showed significant enrichment of the glycolysis gene set between non-pCR and pCR tumors (NES = −1.45, *P* < 0.005; **Figure 4E**), suggesting a potential association between glycolysis-related transcriptional programs and chemotherapy response.

*CCND1* expression positively correlated with pyruvate abundance in both the non-pCR (R = 0.833, *P* < 0.01) and pCR (R = 0.761, *P* = 0.01) groups (**Figures 4F, G**), suggesting a potential link between CCND1 and glycolysis-related metabolic reprogramming. Functionally, wound-healing and Transwell invasion assays verified that CCND1 knockdown markedly attenuated cell migration and invasion, with equivalent inhibitory effects observed at both 24 h and 48 h post knockdown (**Figures 4H–J**). Furthermore, exogenous pyruvate supplementation promoted cell migration and invasion, indicative of a more aggressive malignant cellular phenotype (**Figures 4K–M**).

In summary, our multi-omics analyses identify a glycolysis-associated metabolic state characterized by elevated pyruvate that differentiates non-pCR from pCR BC. Our data support functional interplay between pyruvate and CCND1 and suggest a metabolic–oncogenic axis that may contribute to poor response to NAC, highlighting a potential therapeutic strategy in BC.

### 3.5 Pyruvate activates a pro-tumorigenic transcriptional program centered on MET signaling

**Figure 5:**
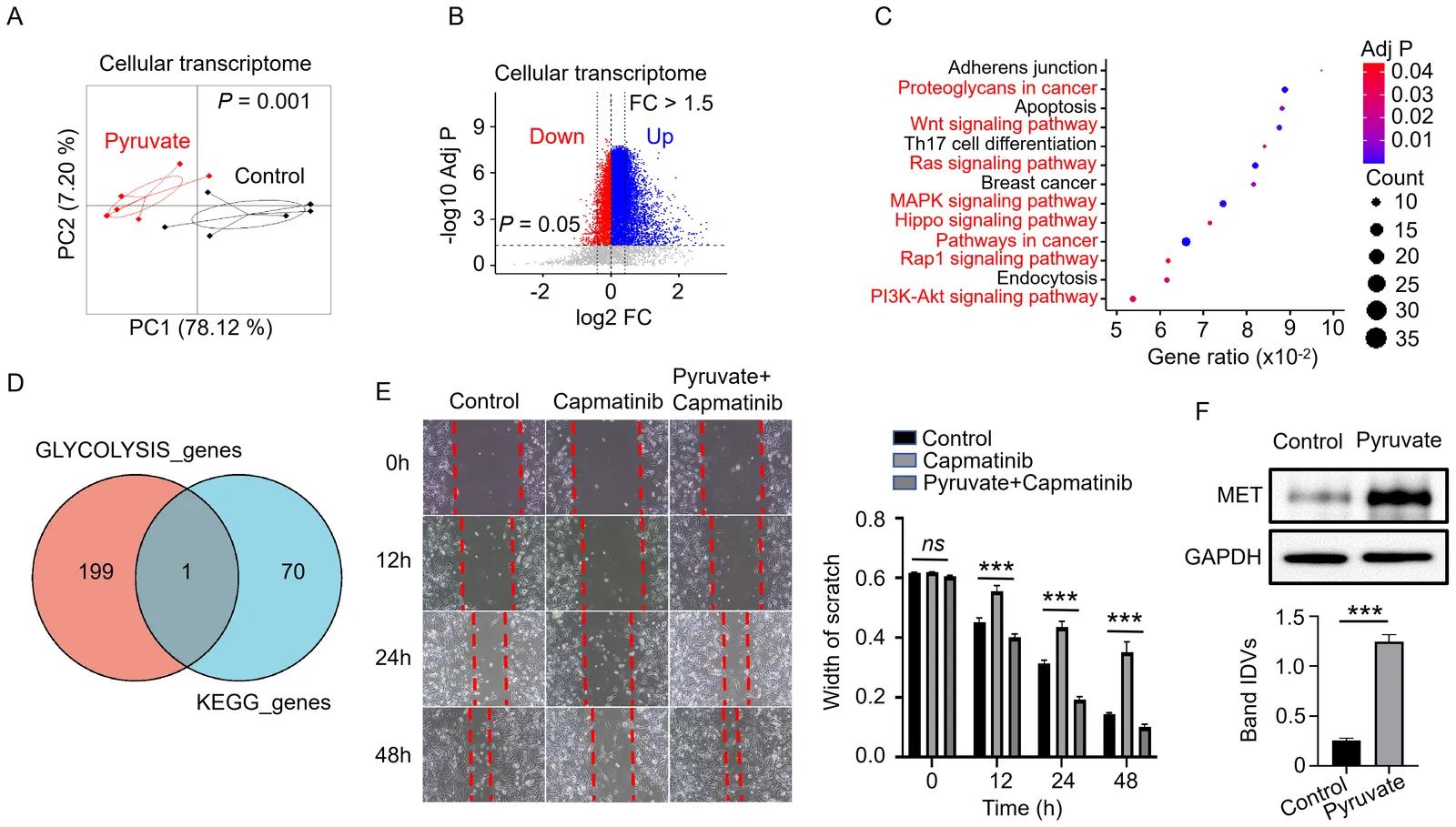
Pyruvate activates MET to promote tumor migration and invasion. **(A)** PCA analysis of the cellular transcriptome. **(B)** Volcano plot displaying DEGs between pyruvate-treated and control samples. **(C)** Significantly enriched KEGG pathways among the DEGs. **(D)** Venn diagram depicting the intersection of glycolysis-related genes and pathway-enriched genes. **(E)** Wound-healing and Transwell assays showed that capmatinib inhibited cell migration, and pyruvate treatment reversed the inhibitory effect of capmatinib. **(F)** Western blot showing that pyruvate promotes MET expression. Results were summarized as mean ± SD of three independent experiments. (* *P* < 0.05; ** *P* < 0.01; *** *P* < 0.001, independent Student’s t-test).

To characterize pyruvate-induced transcriptomic changes, we performed RNA sequencing (RNA-seq) on BC cells after pyruvate treatment. We identified 11,563 DEGs, including 9,300 upregulated and 2,263 downregulated genes after pyruvate treatment (**Figures 5A, B**).

KEGG pathway enrichment analysis of these DEGs was conducted using the KOBAS platform. The analysis showed significant enrichment of oncogenic and therapy-resistance–related pathways, including PI3K–Akt, MAPK, Wnt, and Ras signaling, as well as the Proteoglycans in cancer and Pathways in cancer KEGG pathways (**Figure 5C**). To connect these pathway signals to glycolysis, we intersected pathway-mapped genes with a curated glycolysis-related gene set and identified *MET*, a receptor tyrosine kinase, as the key overlapping gene (**Figure 5D**).

In wound-healing assays, the *MET* inhibitor capmatinib significantly reduced cell migration and proliferation. In contrast, pyruvate supplementation increased migration and proliferation. Notably, capmatinib co-treatment substantially reversed the pyruvate-induced increase in migration, indicating that this effect is MET dependent (**Figure 5E**). Consistently, western blotting showed that pyruvate treatment increased *MET* protein levels, suggesting that MET may act downstream of pyruvate-associated signaling (**Figure 5F**).

In summary, our integrated analyses indicate that pyruvate is associated with induction of a broad pro-tumorigenic transcriptional program. We further implicate the MET signaling axis as a key mediator of pyruvate-associated effects on cancer cell proliferation and migration, and as a potential contributor to reduced therapeutic sensitivity, highlighting a metabolic–transcriptional link in BC.

### 3.6 Single-cell profiling reveals CCND1 distribution and a YAP1–HIF1A–MET regulatory axis in malignant epithelial cells

**Figure 6:**
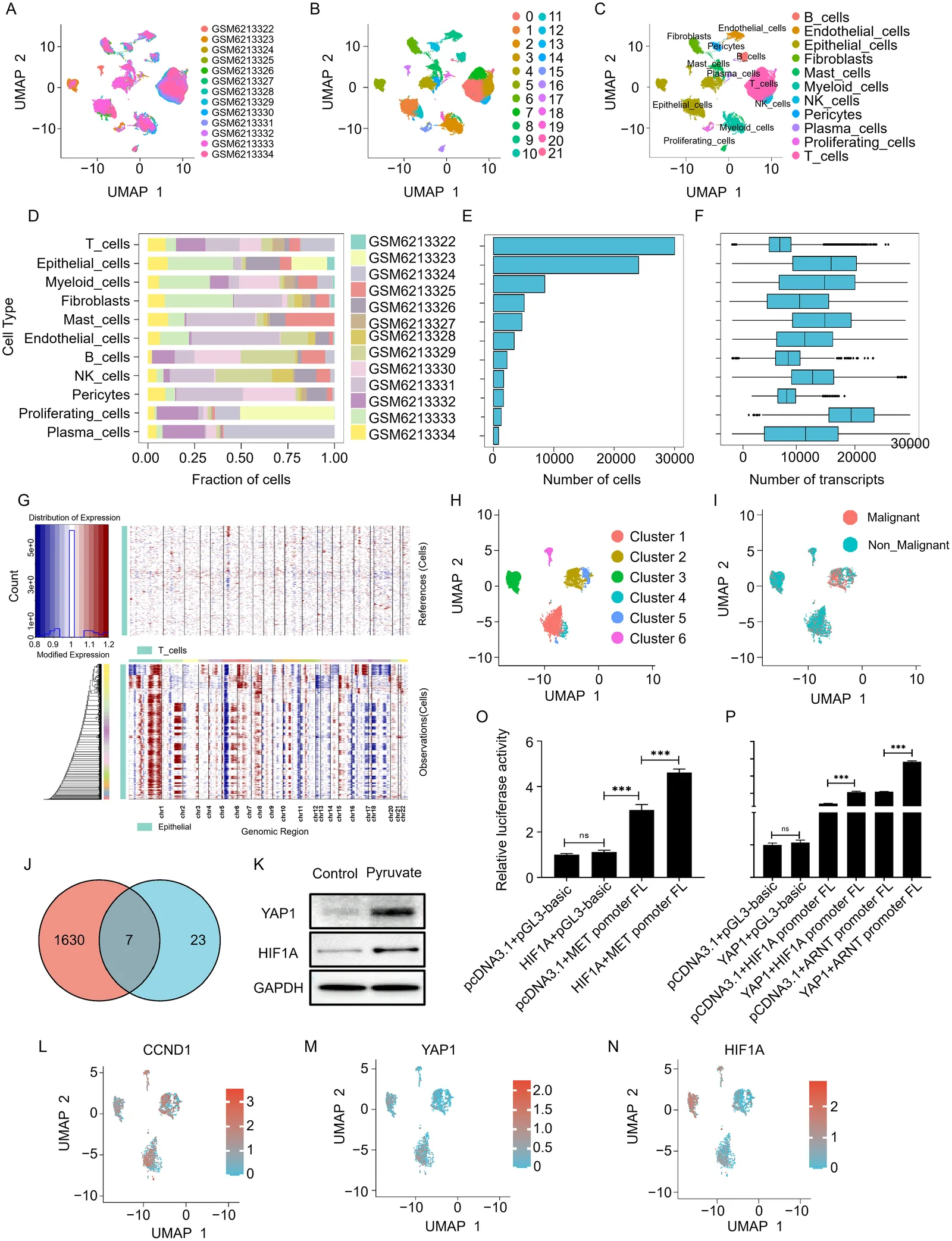
Single-cell RNA-seq landscape of breast cancer and regulatory mechanism of *YAP1*-mediated *HIF1A*/*ARNT*-dependent MET transcription. **(A)** Cellular composition and distribution across 13 patient samples analyzed by scRNA-seq. **(B)** Unsupervised clustering of breast cancer scRNA-seq data reveals 21 distinct cell clusters. **(C)** Annotation of the 21 clusters into 11 major cell types based on canonical marker genes. **(D–F)** Box plots summarizing the proportional abundance **(D)**, absolute cell numbers **(E)**, and transcript counts **(F)** for the 11 major cell lineages across the 13 samples. **(G)** CNVs inferred in epithelial cells using T cells as a reference. Red indicates amplification, and blue indicates deletion. **(H)** UMAP visualization of epithelial cell subclusters, colored by their original cluster identity. **(I)** UMAP plot distinguishing malignant (red) and non-malignant (blue) epithelial cells based on CNV inference. **(J)** Venn diagram depicting the overlapping genes between *CCND1*-correlated genes and transcription factors. **(K)** Western blot showing that pyruvate promotes *YAP1* and *HIF1A* expression. **(L–N)** UMAP plot showing co-expression of *CCND1*, *YAP1* and *HIF1A* in a subset of malignant tumor cells. **(O, P)** Dual-luciferase reporter assays showed that *HIF1A* activates the MET promoter, while *YAP1* transactivates both *HIF1A* and *ARNT* promoters.

To map the cellular composition of BC and examine cell type–specific expression of key genes, we performed single-cell RNA sequencing (scRNA-seq) on 13 tumor specimens from patients receiving NAC. The cohort comprised eight pre-NAC samples (5 non-pCR, 3 pCR) and five post-NAC samples (2 non-pCR, 3 pCR) (**Figure 6A**). After stringent quality control, we retained 83,538 high-quality cells. We performed batch correction with Harmony and then conducted unsupervised clustering, identifying 22 transcriptionally distinct clusters (**Figure 6B**). Cell-type annotation using canonical marker genes identified 11 major lineages: T cells (35.9%), epithelial cells (28.8%), myeloid cells (10.2%), mast cells (6.1%), fibroblasts (5.7%), endothelial cells (4.1%), B cells (2.7%), pericytes (2.0%), NK cells (2.0%), proliferating cells (1.6%), and plasma cells (1.0%) (**Figure 6C**).

The lineage composition and distribution across individual samples, together with absolute cell counts and transcriptional metrics, are presented in (**Figures 6D–F**). Lineage-resolved expression analysis showed that *CCND1* was detected across multiple cell types, including epithelial, endothelial, and fibroblast cells, as well as mast cells, myeloid cells, pericytes, and proliferating cells; expression was highest in epithelial cells (**Figure 6G**). *CCND1* expression was visualized alongside canonical markers for epithelial (*EPCAM*), endothelial (*PECAM1*), and fibroblast (*COL1A1*), myeloid (*CD68*), and T cells (*CD3D*) (**Supplementary Figure 1**). Together, these scRNA-seq results indicate that CCND1 is predominantly enriched in epithelial cells and support its prioritization as a key gene within the glycolysis-associated prognostic signature linked to non-pCR in our earlier analyses.

To further characterize malignant epithelial cells and explore the underlying regulatory mechanism, we integrated scRNA-seq with CNV inference in pre-NAC tumor specimens. Using T cells as a diploid reference, inferCNV identified widespread somatic copy-number alterations predominantly in epithelial cells (**Figure 6G**). Unsupervised clustering of CNV profiles separated epithelial cells into six distinct subpopulations (**Figure 6H**). Annotation indicated that Cluster 2 was the predominant subpopulation enriched for genomically aberrant epithelial cells, consistent with a malignant phenotype (**Figure 6I**).

To identify transcriptional programs associated with the malignant epithelial phenotype, we performed co-expression analysis in malignant epithelial cells and identified 1,637 genes positively correlated with *CCND1*. Intersecting this gene set with a curated transcription factor list from GeneCards yielded seven candidate regulators: *MYC, E2F1, YAP1, CTNNB1, FOXO3, SOX9*, and *HIF1A* (**Figure 6J**). *YAP1* activity is tightly controlled by the Hippo pathway; as a quintessential mechanosensor, it responds to extracellular matrix (ECM) stiffness, cell morphology and cell density to further modulate tumor progression (20). *HIF1A*, on the other hand, is closely linked to glycolysis and regulated via oxygen-dependent protein stabilization (21). Western blotting showed that pyruvate treatment significantly increased *YAP1* and *HIF1A* protein levels (**Figure 6K**). Single-cell expression mapping further demonstrated co-enrichment of *CCND1, YAP1*, and *HIF1A* within the malignant epithelial subpopulation (**Figures 6L–N**).

We further interrogated the regulatory hierarchy using dual-luciferase reporter assays. *HIF1A* overexpression significantly increased *MET* promoter activity (**Figure 6O**). *YAP1* overexpression significantly increased the promoter activities of *HIF1A* and *ARNT* (**Figure 6P**). Together, these results support a *YAP1–HIF1A–MET* transcriptional axis in which *YAP1* enhances *HIF1A* and *ARNT* transcriptional activity, thereby promoting *MET* transcription. Pyruvate-associated activation of this axis provides a mechanistic link between glycolysis-related metabolic changes and transcriptional programs that promote pro-tumorigenic signaling in BC.

## 4 Discussion

This study addresses a central clinical challenge in BC: why some patients achieve pCR after NAC while others develop non-pCR residual disease (22). We established an evidence chain that connects a pretreatment predictive molecular score to an interpretable mechanistic framework. Our results indicate that non-pCR is not a single-pathway resistance phenomenon. Instead, it represents a broader state of reduced treatment sensitivity that can be jointly described by tumor-intrinsic programs and the surrounding cellular milieu. Specifically, enhanced proliferative drive and adaptive signaling within tumor cells, together with metabolic shifts and microenvironment-associated programs, act in concert to increase the likelihood and persistence of residual disease.

From a clinical usability perspective, our study shows that integrating transcriptomic data across multiple cohorts can recover differential signals that are consistent across datasets. Using LASSO regression, we distilled these signals into a 15-gene signature and a corresponding RS. The RS maintained strong discriminatory performance in both the training set and external validation cohorts, with stable AUC values across datasets. It also showed a consistent association with pathologic response in regression analyses, supporting its potential value for risk stratification. Notably, the RS is more than a purely statistical classifier: it condenses complex resistance-related molecular changes into a small set of interpretable gene weights, providing a clear molecular entry point for downstream mechanistic validation (23).

Consistent with the molecular differences captured by the RS, our data indicate that non-pCR tumors display a stronger combined pattern of proliferation, inflammation, and hormone responsiveness (24). Differential expression and gene set enrichment analyses converged on increased cell-cycle progression (for example, E2F-associated programs and the G2/M checkpoint) and heightened epithelial proliferation and developmental processes (25). In parallel, inflammatory signaling (such as TNFα/NF-κB) and estrogen-response programs were also enriched, implicating broader communication networks including JAK–STAT signaling and cytokine–receptor interactions (26). Taken together, this transcriptional profile supports a more integrated interpretation. When we grouped pretreatment samples by post-NAC outcome (pCR vs non-pCR), the non-pCR group already showed baseline features consistent with an adaptive, chemotherapy-tolerant state—combining a cell-cycle–driven capacity for regrowth with inflammatory and intercellular signaling programs that may promote survival and tissue remodeling (27).

Previous studies concerning CCND1 in BC predominantly focused on its canonical roles in cell cycle progression and tumor proliferation (28). Distinct from these researches, our study has two key innovations. Clinically, we constructed a pretreatment 15-gene risk signature to predict non-pCR outcomes in BC patients receiving NAC, providing a novel tool for clinical risk stratification. Mechanistically, by combining transcriptomics, serum metabolomics, single-cell RNA-seq and *in vitro* functional experiments, we identified a novel pyruvate–CCND1–MET regulatory axis driving NAC resistance, as well as the upstream YAP1–HIF1A/ARNT transcriptional cascade. This study links *CCND1* function to glycolytic metabolism and receptor tyrosine kinase signaling, which greatly expands the understanding of CCND1 and complements existing research in this field. TNBC represents one of the predominant molecular subtypes eligible for neoadjuvant chemotherapy owing to its high metastatic potential and limited targeted therapeutic options. MDA-MB-231 is a well-established TNBC cell line, which is widely used to investigate BC malignant phenotypes and chemoresistance (29). Our multi-omics analyses verified that the aberrant activation of CCND1, glycolysis and MET is a common molecular trait of NAC-resistant BC across all molecular subtypes. Therefore, we adopted this classic cell model for *in vitro* functional validation, and the cellular phenotypes and molecular alterations observed in cell experiments are consistent with the molecular profiles of NAC-resistant tumors from patient cohorts.

Accumulating evidence has confirmed that these four core genes serve as classic biomarkers across multiple human cancers, with well-characterized roles in tumor diagnosis, prognosis and treatment response prediction (28, 30). As a key cell cycle regulator, *CCND1* overexpression and amplification are widely reported in BC, and multiple studies have verified its correlation with NAC resistance and adverse clinical outcomes (13). Beyond BC, elevated CCND1 expression also acts as an independent poor prognostic factor in gynecological tumors such as vulvar squamous cell carcinoma, revealing its universal oncogenic function across epithelial malignancies (31). *ESR1*, the estrogen receptor alpha gene, is a routine clinical biomarker for molecular subtyping of BC; its expression status directly guides endocrine therapy and neoadjuvant treatment strategies (32). Aberrant ESR1 expression is also frequently detected in ovarian, endometrial and other hormone-related cancers, indicating broad diagnostic value in hormone-dependent tumors (33).

*TSPAN1*, a member of the tetraspanin family, has been reported to drive tumor proliferation and metastasis in various solid tumors (34). Dysregulated TSPAN1 expression has been associated with tumor progression and chemotherapy resistance in several malignancies, whereas its potential value for predicting NAC response in BC remains less well defined (34). *CCL18*, a chemokine secreted mainly by M2-type macrophages, is a critical biomarker of the tumor immune microenvironment (35). In BC, high CCL18 expression correlates with M2 macrophage infiltration and inferior NAC efficacy (36). Furthermore, elevated CCL18 has been associated with diagnosis or prognosis in non-small-cell lung cancer, glioma, and diffuse large B-cell lymphoma, supporting its broader relevance to immunosuppressive tumor microenvironments and tumor progression across malignancies (37).

In general, our findings are consistent with the oncogenic properties of these four genes described in previous pan-cancer studies. Distinct from prior research that mainly focused on tumor diagnosis or general prognosis, our work further links this four-gene panel specifically to NAC non-pCR in BC. Among them, CCND1 stands out as the most prominent hub gene in our multi-omics analysis, which is in line with its established role in regulating cell cycle progression and chemotherapy resistance in both BC and other tumor types. Collectively, these multilayer analyses define a concise four-gene signature that discriminates non-pCR from pCR BC and may have clinical utility. Among these genes, CCND1 is a promising candidate for predictive biomarker development and a potential therapeutic target.

At the level of key nodes, our multi-omics integration and layered validation consistently identified *CCND1* as a prominent hub. By intersecting our results with gene sets linked to tumorigenesis, we distilled a core gene panel—*CCND1, TSPAN1, ESR1*, and *CCL18*—that distinguishes non-pCR from pCR. Among these, *CCND1* showed the most pronounced upregulation in non-pCR tumors, suggesting that it may sit near the center of the network underlying reduced treatment sensitivity. This finding aligns biologically with the heightened cell-cycle and proliferation programs observed in non-pCR and provides a clear direction for probing upstream drivers (25). In particular, increased capacity for cell-cycle progression may be a key foundation for the persistence and expansion of residual disease (38).

Our data further indicate that metabolic reprogramming provides a measurable, system-level support for the hub network described above. Serum metabolomics revealed marked metabolic differences between non-pCR and pCR, with glycolysis/gluconeogenesis pathways standing out. Pyruvate levels were elevated in patients overall and were higher in the non-pCR group; importantly, pyruvate abundance showed a strong positive correlation with *CCND1* expression. Functional experiments supported the phenotypic consequences of these associations: *CCND1* knockdown reduced cell migration and invasion, whereas pyruvate supplementation enhanced these behaviors. Together, these results suggest that pyruvate may serve not only as a response-associated metabolic marker but also as an active contributor to invasive traits linked to poor treatment outcomes.

At the mechanistic effector level, our results suggest that pyruvate can convert metabolic change into pro-tumor behavior through the MET signaling axis. Transcriptomic profiling after pyruvate treatment revealed widespread expression changes and enrichment of multiple cancer- and therapy-tolerance–related signaling networks; integrative analyses identified *MET* as a key hub. Crucially, the *MET* inhibitor capmatinib significantly blunted the pyruvate-induced increases in proliferation and migration, and protein-level assays showed that pyruvate upregulated *MET* expression. Together, these findings connect metabolic state, a druggable target, and cellular behavior into a testable functional chain, providing a clear rationale and experimental support for combination intervention strategies (39).

Single-cell evidence further strengthens the plausibility of this pathway by clarifying where the signal resides. Our scRNA-seq data detected *CCND1* across multiple lineages, but its highest expression was in epithelial cells, supporting a primary association with the tumor epithelial/malignant compartment. With finer resolution within malignant epithelial cells, we identified a putative transcriptional regulatory cascade linking *YAP1, HIF1A* (with its partner *ARNT*), and *MET*. Specifically, pyruvate increased *YAP1* and *HIF1A* protein levels; HIF1A could activate the MET promoter; and YAP1 could promote transcription from the HIF1A and ARNT promoters. This regulatory structure offers a mechanistic explanation for how a metabolic cue can upregulate MET and thereby promote pro-tumor programs (40). By proposing this axis, the pyruvate-associated effects move beyond correlation and pathway enrichment toward a clearer, testable causal framework at the molecular level.

Based on these findings, we propose that non-pCR represents a treatment-insensitive phenotype shaped by three connected components: (i) reinforcement of a cell-cycle hub, exemplified by *CCND1*; (ii) shifts in metabolic substrate availability and glycolytic state, reflected by elevated pyruvate; and (iii) downstream execution through transcriptional control and receptor signaling along the *YAP1/HIF1A–ARNT–MET* axis. This model is also compatible with the enhanced inflammatory and cytokine-related networks observed in transcriptomic analyses. Heightened metabolic and receptor signaling may further amplify intercellular communication and remodel the microenvironment, thereby creating a niche that supports residual disease. However, the extent to which inflammatory and immune lineages play a decisive role in this framework remains to be defined with more direct functional and spatial evidence (38).

Several limitations of this study also define clear priorities for future work. Although the 15-gene RS performed consistently across cohorts, real-world factors—such as molecular subtype (ER/HER2/TNBC), treatment regimens, and differences in sampling and assay platforms—may affect calibration and generalizability (41). Prospective, multicenter validation with subtype-stratified adjustment will be required to establish clinical utility and to define the most appropriate use cases (for example, pretreatment stratification, early response monitoring, or residual-risk assessment) (42). The metabolomics analysis was based on a relatively limited sample size, and serum pyruvate levels may be influenced by diet and metabolic comorbidities (43). Future studies should implement tighter sampling control and repeated measurements, and test whether joint modeling of pyruvate with the RS or key genes can further improve predictive performance and robustness (44). Mechanistic evidence currently relies mainly on *in vitro* systems and pharmacologic inhibition. While our data support a pyruvate–MET–dependent functional link and a transcriptional mechanism involving the YAP1/HIF1A (ARNT)–MET axis, organoid, PDX, and animal studies are still needed to establish *in vivo* causality for chemotherapy sensitivity and residual-disease formation, and to evaluate potential benefit windows and toxicities when combined with NAC (45).

Overall, our study introduces a quantifiable entry point for risk stratification through a 15-gene RS and, through multi-omics and mechanistic data, supports an integrated metabolism–transcription–signaling axis associated with non-pCR. Elevated pyruvate is coupled to the CCND1 hub and promotes invasive phenotypes; upstream regulation may involve YAP1 and HIF1A (with ARNT), and downstream effects converge on MET signaling. This framework provides a continuous line of evidence for how residual disease may emerge after chemotherapy and establishes a foundation for combination strategies and prospective validation focused on metabolic state and the MET pathway. Additionally, we acknowledge a key limitation of this study: *MET* functions as a central signaling hub intersecting multiple conserved proliferative, metabolic and stress-response pathways, so systemic MET inhibition may elicit diverse unforeseen downstream effects in non-malignant tissues (46, 47). Although published genome-wide transcriptional and protein regulatory networks (STRING, Reactome, MSigDB) enable in silico prediction of broad molecular perturbations triggered by MET blockade, comprehensive network-based simulation of full systemic drug impacts was not performed in the current work, as our core experimental design prioritized establishing a pretreatment predictive signature and dissecting tumor-specific pyruvate-driven chemoresistance mechanisms (48, 49). Follow-up research will integrate curated human regulatory interactomes and quantitative systems pharmacology pipelines to computationally predict the complete downstream transcriptional cascade induced by MET suppression, and further validate both therapeutic benefits and potential toxic off-target phenotypes using patient-derived organoids and animal models to refine the translational safety profile of MET-targeted combinatorial therapy for NAC-resistant BC.

## Data Availability

All public transcriptomic datasets (GSE25066, GSE20194, GSE32646, GSE205472) are deposited in the GEO repository with accessible accession IDs. The custom analysis code is available at [https://github.com/chenjinhao-nb/CCND1-paper-code](https://link.wtturl.cn/?target=https%3A%2F%2Fgithub.com%2Fchenjinhao-nb%2FCCND1-paper-code&scene=im&aid=497858&lang=zh). Clinical raw metabolomic and transcriptomic data cannot be publicly shared due to patient privacy restrictions, and reasonable requests for these datasets can be sent to the corresponding author.
