# Supplementary material for: A pretreatment 15-gene signature predicts non-pCR in response to neoadjuvant chemotherapy in breast cancer and reveals a pyruvate–CCND1–MET therapeutic vulnerability
Source: Front Oncol. 2026 Aug 6;16:1829672. doi: 10.3389/fonc.2026.1829672 (PMC13489880; doi:10.3389/fonc.2026.1829672)
Supplement: Supplementary file 1 [file DataSheet1.docx]

**Supplementary Figure S1. Marker gene expression profiles for cell type annotation in single-cell RNA-seq data.** (A) Expression patterns of representative marker genes used for cell type identification.
